# Toxicological Assessment of an Acrylic Removable Orthodontic Appliance Using 2D and 3D In Vitro Methods

**DOI:** 10.3390/ma15031193

**Published:** 2022-02-04

**Authors:** Stefania Dinu, Emanuela Lidia Craciunescu, Ioana Macasoi, Doina Chioran, Mircea Rivis, Daliborca Vlad, Raluca Adriana Milutinovici, Iasmina Marcovici, Alina Dolghi, Alina Moaca, Dorin Cristian Dinu, Cristina Dehelean, Malina Popa

**Affiliations:** 1 Department of Pedodontics, Faculty of Dental Medicine, Victor Babes University of Medicine and Pharmacy, 9 No., Revolutiei 1989 Bv., 300041 Timisoara, Romania; dinu.stefania@umft.ro (S.D.); popa.malina@umft.ro (M.P.); 2 Pediatric Dentistry Research Center, Faculty of Dental Medicine, Victor Babes University of Medicine and Pharmacy, 9 No., Revolutiei Bv., 300041 Timisoara, Romania; 3 Department of Prostheses Technology and Dental Material, Faculty of Dental Medicine, Victor Babes University of Medicine and Pharmacy, 9 No., Revolutiei 1989 Bv., 300041 Timisoara, Romania; emanuela.craciunescu@umft.ro; 4 Romania Research Center in Dental Medicine Using Conventional and Alternative Technologies, Faculty of Dental Medicine, Victor Babes University of Medicine and Pharmacy, 9 No., Revolutiei Bv., 300041 Timisoara, Romania; 5 Department of Toxicology and Drug Industry, Faculty of Pharmacy, Victor Babes University of Medicine and Pharmacy, 2nd Eftimie Murgu Sq., 300041 Timisoara, Romania; macasoi.ioana@umft.ro (I.M.); iasmina.marcovici@umft.ro (I.M.); dolghi.alina@umft.ro (A.D.); alina.moaca@umft.ro (A.M.); cadehelean@umft.ro (C.D.); 6 Research Center for Pharmaco-Toxicological Evaluations, Faculty of Pharmacy, Victor Babes University of Medicine and Pharmacy, Eftimie Murgu Square No. 2, 300041 Timisoara, Romania; 7 Department of Dento-Alveolar Surgery, Faculty of Dental Medicine, Victor Babes University of Medicine and Pharmacy, 9 Revolutiei 1989 Ave., 300070 Timisoara, Romania; 8 Department of Anesthesiology and Sedation in Dentistry, Dento-Alveolar Surgery, Faculty of Dental Medicine, Victor Babes University of Medicine and Pharmacy, 2nd Eftimie Murgu Sq., 300041 Timisoara, Romania; 9 Department of Pharmacology and Biochemistry-Pharmacology, Faculty of Medicine, Victor Babes University of Medicine and Pharmacy, 2nd Eftimie Murgu Sq., 300041 Timisoara, Romania; vlad.daliborca@umft.ro; 10 Department of Orthodontics, Faculty of Dental Medicine, Victor Babes University of Medicine and Pharmacy, 9 Revolutiei 1989 Ave., 300070 Timisoara, Romania; raluca_balan22@yahoo.com; 11 Orthodontic Research Center (ORTHO-CENTER), Faculty of Dental Medicine, Victor Babes University of Medicine and Pharmacy, Revolutiei Ave. 1989 No. 9, 300041 Timisoara, Romania; 12 Family Dental Clinic, Private Practice, 24 Budapesta Str., 307160 Dumbravita, Romania; dorin@dr-dinu.com

**Keywords:** acrylic removable appliance, orthodontics, in vitro, 3D reconstructed human epidermis, biocompatibility, cytotoxicity

## Abstract

Malocclusion is a global health problem, mainly affecting children and adolescents. For this reason, orthodontic treatment must be, on the one hand, safe, non-toxic, and effective and, on the other hand, it must have the best possible esthetic profile. Thus, the use of orthodontic appliances is addressed to all age groups, including young children, for a long period of time, which is why their safety profile is a matter of real interest. For this reason, the purpose of the present study was to evaluate the safety and biocompatibility of an acrylic removable orthodontic appliance made of polymethylmethacrylate and stainless steel alloy made by our team of researchers. To verify the biocompatibility of the medical device, it was immersed in artificial saliva with three different pHs (3, 7, and 10) for a period of ten days. Subsequently, the three types of saliva were tested on human keratinocytes (HaCaT cell line) in terms of viability and modification of cell morphology. Finally, the use of 3D reconstructed human epidermis verified the cytotoxic and irritating potential of the medical device, thus providing relevant information regarding its biocompatibility. The results revealed that by maintaining the orthodontic device in the saliva there is no release of substances with a toxic effect on the human keratinocytes and on the 3D reconstructed human epidermis. There were also no significant changes in cell morphology. In conclusion, it is suggested that the acrylic removable appliance has a safety profile recommended for in vivo use.

## 1. Introduction

Despite not being considered a disease in itself, malocclusion is considered to be a global health problem. The development of orthodontic treatment to treat malocclusion, having a significant impact on the quality of life, has gained real interest from health professionals [1]. A complete definition of malocclusion involves the irregular growth of the teeth or a defective relationship between the dental arches beyond normal limits. Apart from having an impact on chewing, speech, and dental esthetics, this health issue also affects the person’s psychological state [2]. The occurrence of malocclusion is based on both genetic factors and environmental factors related to the baby’s nutrition [3]. 

In view of the fact that malocclusion occurs in childhood, orthodontic treatment must be safe, in the sense of toxicity, and patient compliance must be high, given that it is a long-term treatment [4]. The main concern of specialists in the field is the development of orthodontic appliances that combine both appropriate esthetic characteristics and technical performance necessary for effective treatment. One of the therapeutic options for malocclusion is to use smaller brackets made of stainless steel [5], but, although they are therapeutically effective, they do not meet the esthetic criteria [6]. On the other hand, lingual orthodontics, another therapeutic approach to malocclusion, have the advantage of being esthetic, but they are not as effective and raise several problems for both therapist and patient [7]. Another alternative to fixed orthodontic appliances is clear aligner systems. These are divided into two categories, depending on how they are obtained, as follows: (i) devices made of thermoplastic materials by manual set-up; and (ii) aligns produced by CAD-CAM technology [8]. An example of clear aligner systems is the Clear Aligner CA System (Scheu Dental, Germany) [9]. This type of orthodontic device offers multiple advantages, such as the easy manufacture of alignment in laboratory conditions in a cost-effective manner and the facilitation of the treatment follow-up process and the intervention of the orthodontist to make necessary changes in treatment [8]. Invisalign, one of the most well-known and used clear aligner systems, belongs to the category of aligners made by CAD-CAM technology [10]. This type of orthodontic device has the advantage of treating mild cases without extraction faster, but, nevertheless, studies have shown that it requires a longer time than fixed appliances for the treatment of more complex cases [11]. Although these types of devices offer a good esthetic to patients, they have several disadvantages, such as long treatment time, risk of resorption, or biocompatibility problems due to the materials from which they are made [12].

Depending on the severity of the condition, orthodontic therapy includes two main approaches, namely, the use of fixed or detachable appliances [13]. Although conventional orthodontic fixed appliances have proven useful in the treatment of malocclusion, they are not without side effects. The most common problems that can occur during orthodontic treatment include changes in oral pH and the development of cavities, gingivitis, and periodontitis [14]. The main cause of these side effects is poor hygiene of orthodontic patients. In this case, the type of orthodontic appliance used, as well as the bracket material, the bonding technique, and also the treatment time, are key factors that can influence the realization of proper oral hygiene [15].

Removable orthodontic appliances have been in medical practice for a long time. Although studies have shown that these types of orthodontic appliances are not as effective as fixed ones, they are still in therapeutic use for several dental problems, such as overbite reduction, tipping teeth, or block movements [16,17]. 

Researchers have focused their attention on the development of new orthodontic appliances that are made from materials with improved mechanical properties and do not expose patients to the risk of releasing bisphenol A (BPA). BPA is a toxic compound with a systemic impact on the body, especially in the endocrine system [18]. BPA toxicity in the endocrine system is mainly due to the ability of this substance to interact with various receptors, such as the estrogen receptor, the androgen receptor, and the thyroid hormone receptor [19]. In addition to the harmful effects of BPA on various systems, such as the reproductive [20] or nervous systems [21], recent studies have suggested a correlation between BPA exposure and tumor development [22]. In vitro studies have shown that BPA stimulates the viability of breast cancer cells and increases oxidative stress [23]. Regarding the release of BPA from orthodontic appliances, it was observed that the maximum measured BPA release values, both in vitro and in vivo, were recorded in the first week after the implementation of the biomaterial [23].

One of the most widely used materials for making acrylic removable orthodontic appliances is polymethylmethacrylate (PMMA) [17]. PMMA (IUPAC name: poly [1-(methoxy carbonyl)-1-methyl ethylene]) is a synthetic polymer made by a polymerization reaction of methyl methacrylate (C_5_O_2_H_8_) to polymethyl methacrylate (C_5_O_2_H_8_)_n_. The polymerization reaction can be initiated either chemically or with energy. Depending on the mode of initiation of the polymerization reaction, there are three main types of polymers (I, II, and III), which differ from each other depending on the reactions and polymerization compositions. In addition to these three main types of PMMA, two more were included, type IV (light-activated polymers) and type V (microwave-activated polymers) [24]. PMMA must also have favorable physical properties for orthodontic applications. One of the most important physical properties is the absorption of water or other oral fluids. This is due to the molecular polarity, so that water molecules can infiltrate the polymer chains and act as plasticizers [25]. Another property worth considering is solubility. This can affect dimensional stability. Thus, both the absorption of liquids and the associated solubility must be controlled and kept to a minimum [24].

The main features of PMMA useful for applications in dentistry and orthodontics are its low density and remarkable physical and mechanical properties [26]. While using a medical device, biocompatibility is of paramount importance. Regarding PMMA, studies have suggested that this type of material has good biocompatibility [27,28,29]. However, due to errors during manufacture, it can lead to the presence of hardened or residual monomers responsible for the inflammation and irritation of the oral mucosa [24]. In addition, these errors can affect other important properties of the material, such as decreased mechanical properties, discoloration, and even the appearance of allergic reactions [17].

Since it is crucial that materials used in orthodontics be biocompatible and have the right properties to be effective, obtaining PMMA is of paramount importance. Thus, several methods for obtaining dental polymers have been described in the literature. One method is to microwave postpolymerize the resins to align the dental base [30]. In addition, in the literature there has been described the method of postcuring by means of electron beam or heat postpolymerization [24], both methods improving the mechanical properties of the obtained material. Another approach to reducing unreacted monomers is to apply secondary heat treatment, but this method has the disadvantage that it can cause color changes during the process [17].

A secondary concern with conventional orthodontic appliances is their metal parts, which are often comprised of stainless steel or nickel–titanium alloys. These materials allow the release of metal ions, mainly chromium and nickel ions that can be absorbed by the enamel or can reach the gastrointestinal tract through saliva [31]. In patients with poor oral hygiene, plaque may build up on the vestibular surface of the teeth around the brackets, causing demilitarization of the enamel. In this case, there is the biodegradation of brackets and the release of metal ions that can enter the tooth enamel, a process called metallosis [32]. Regarding the absorption of ions by enamel, Keinan et al. [33] observed that nickel is absorbed in a significantly smaller amount by intact teeth, compared to absorption from saliva. Studies have shown that by absorbing metal ions in the enamel they cause local side effects, such as discoloration of the enamel. Instead, by releasing ions into saliva, they can reach the gastrointestinal tract and cause toxic reactions at the systemic level [31].

For this reason, research directions in the field of orthodontics have focused on the development of innovative orthodontic appliances with improved biological and mechanical properties. One of the recent approaches to orthodontic treatment is the use of acrylic appliances. The materials from which these orthodontic devices are made have the advantage of easy handling and, in addition, have mechanical properties that have attracted the attention of specialists in the field [34]. 

Thus, the present study focused on the development of a removable acrylic orthodontic appliance (ARA). The present study was performed using both 2D and 3D in vitro methods to provide a more comprehensive picture of the biocompatibility of the medical device. 

## 2. Materials and Methods

### 2.1. Reagents

The sample (ARA) was fabricated by Family Dental Clinic (Dumbravita, Romania) in collaboration with Docs Lab from Timisoara, Romania. The following reagents were used for cell culture: phosphate saline buffer (PBS), trypsin-EDTA solution, dimethyl sulfoxide (DMSO), fetal bovine serum (FBS), penicillin/streptomycin, and Resazurin reagent, which were purchased from Sigma Aldrich, Merck KgaA (Darmstadt, Germany), and the cell culture medium, Dulbecco’s Modified Eagle Medium (DMEM–ATCC^®^ 30-2002™), was purchased from ATCC (American Type Cell Collection, Lomianki, Poland). 

All reagents were of analytical grade of purity and for cell culture use.

The following reagents were used to prepare the artificial saliva according to the previous article [35]: CaCl_2_ × 2H_2_O (purity > 99.5%) from Honeywell Fluka™ (Charlotte, NC, USA); NaCl (purity > 99.5%) from Chimopar S.A (Bucharest, Romania); CO(NH_2_)_2_ (purity > 99.5%) from Sigma-Aldrich (St. Louis, MO, USA); KCl (purity > 99.8%), NaOH pellets (purity > 99.3%) from Chimreactiv (Bucharest, Romania); and 37% HCl from Honeywell Fluka^TM^ (Charlotte, NC, USA).

### 2.2. Development of Acrylic Removable Appliance (ARA)

**Figure 1:**
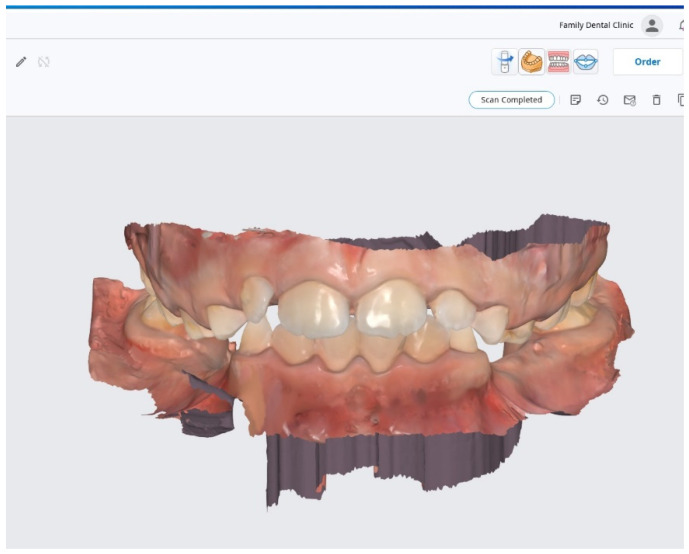
Digital scans of the maxillary and mandibular arches and the bite registration.

**Figure 2:**
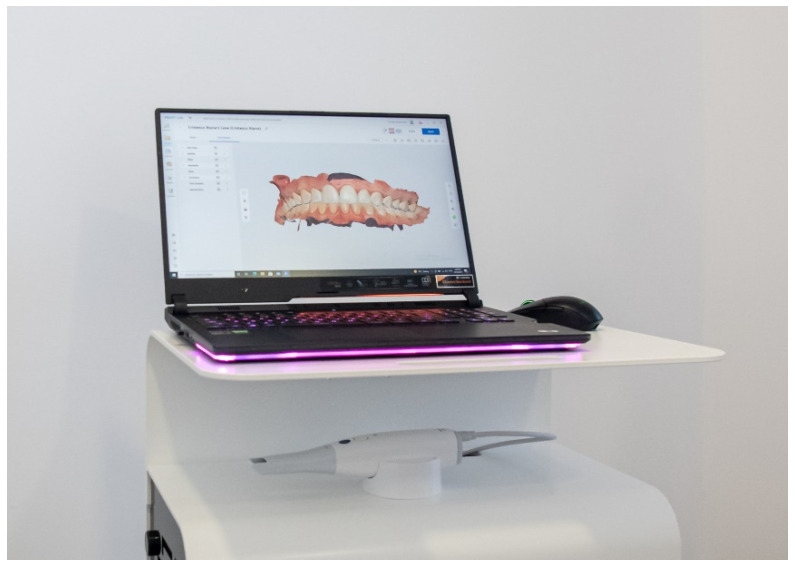
Intraoral scanner MEDIT i700 (Family Dental Clinic).

The orthodontic device used in this study is an acrylic removable appliance made by Docs Lab from Timisoara, Romania. The model cast was made of 3D printing UV-sensitive resin Anycubic with a LCD-based SLA 3D Printer Photon-S printer (HONGKONG ANYCUBIC TECHNOLOGY CO., LIMITED, Shenzhen, China), based on the digital impression (Figure 1) which was taken with the latest and the most advanced 3D intraoral scanner, MEDIT i700 (Medit corp, Seoul, Korea) (Figure 2) in Family Dental Clinic. 

The base plate of the removable appliance was made of polymethylmethacrylate (PMMA, Orthocryl^®^). Orthocryl^®^ was prepared according to the adapting technique, and so the powder and monomer liquid (2.5 parts powder to 1 part liquid) were mixed. Upon reaching the ideal consistency, it was quickly removed, adapted onto the model, and manually molded quickly to the desired shape. Curing was completed in a pressure pot at 2.2 bar, 40 °C for 15 min.

The metal clamps were made of LEOWIRE round spring hard wire C0400-07 and C0400-08 and stainless steel alloy, made to prevent any nickel allergic reactions (Cr 16.5%, Mn 11%, Mo 2.7% N 0.5%, balance: Fe) produced by DENTAURUM GmbH & Co. KG, (Ispringen, Germany).

**Figure 3:**
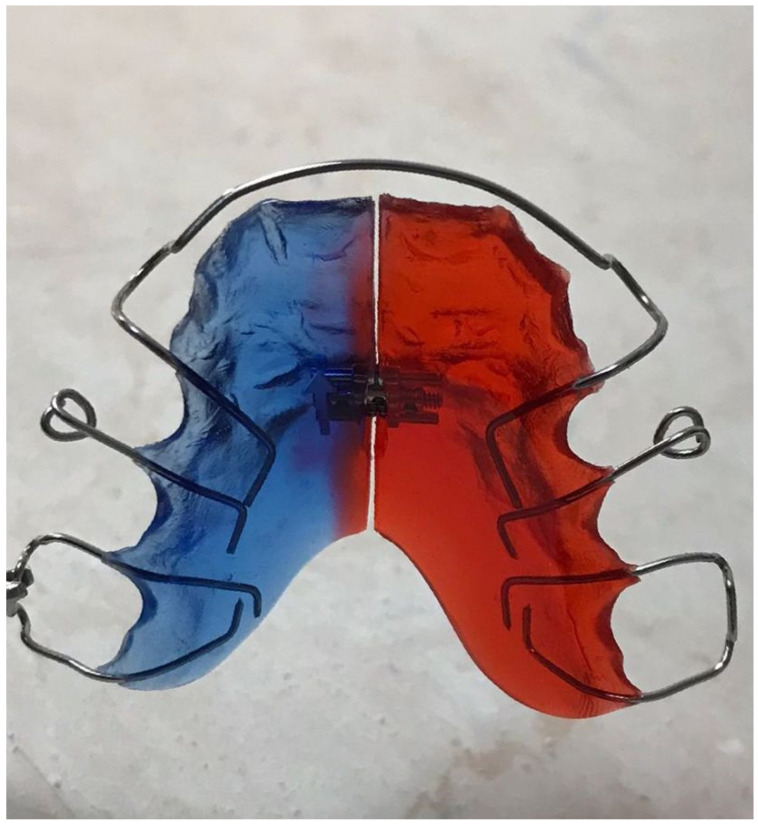
Picture of the acrylic removable appliance (ARA) made by Docs Lab from Timisoara.

The principal screw used was a standard expansion screw with expansion stop and a drill for friction control also produced by DENTAURUM GmbH & Co. KG, (Ispringen, Germany). Figure 3 shows acrylic removable appliance (ARA) obtained.

### 2.3. Preparation of the Artificial Saliva 

Artificial saliva was prepared with three different pHs as follows: (i) acid—3 (at 30.7 °C); (ii) neutral—7 (at 29.6 °C); and (iii) basic—10 (at 29.5 °C). The method of preparation has been described above [35]. To obtain saliva with neutral pH, the following reagents were dissolved in distilled water: 0.40 mg/L NaCl, 0.40 mg/L KCl, 0.80 mg/L CaCl_2_ × 2H_2_O, and 1 mg/L CO(NH_2_)_2_. Finally, 37% HCl was used to obtain the acidic artificial saliva, and 10N NaOH was used to obtain the basic artificial saliva. A Thermo Scientific Eutech pH 150 portable pH meter with the electrode (Thermo Scientific, Waltham, MA, USA) was used to determine the pH of the solutions.

### 2.4. Maintaining the Acrylic Removable Appliance in Artificial Saliva

The orthodontic device was fragmented into three different fragments using a hard wire cutter and acrylic burs for the base plate. Then, the three fragments were immersed in the artificial saliva (acidic, basic, and neutral) and maintained for ten days in an incubator with an orbital shaker (ES20/60 Biosan, Riga, Latvia) at 37 °C. Following this period, the ARA was removed from the saliva, and the three types of saliva (ARAa—acidic saliva; ARAn—neutral saliva; and ARAb—basic saliva) were subsequently used in in vitro tests to assess the device’s cytotoxicity and biocompatibility.

### 2.5. Cell Culture

The present study was performed using human immortalized keratinocytes, the HaCaT cell line, purchased from CLS Cell Lines Service GmbH (Eppelheim, Germany) as a frozen vial. The cells were cultured in their specific medium (DMEM) supplemented with 10% FCS and 1% antibiotics (100 U/mL penicillin/100 µg/mL streptomycin) and incubated in a humidified atmosphere (37 °C, 5% CO_2_) during the experiments.

### 2.6. Cytotoxicity Assessment

The cytotoxic effect of ARA was assessed by means of the 3-(4,5-dimethylthiazol-2-yl)-2,5-diphenyltetrazolium bromide (MTT) viability assay according to the technique described above [36]. In brief, HaCaT cells were seeded in 96-well plates (1 × 10^4^ cells/well) and treated for 24 h and 72 h with five dilutions (1:16, 1:8, 1:4, 1:2, 1:1) of the three types of saliva in which ARA was kept. At the end of the stimulation period, 100 µL of fresh media and 10 µL of MTT reagent were added to each well, followed by a 3 h incubation of the plate at 37 °C. Finally, 100 µL of solubilization solution were added, the plate was incubated for 30 min at room temperature, protected from light, and the absorbance values were read using Cytation 5 (BioTek Instruments Inc., Winooski, VT, USA). The results obtained were expressed as a percentage (viable cells%).

### 2.7. Cell Morphology and Confluence Evaluation

The morphology and confluence of HaCaT cells were evaluated by photographing the cells at the end of the 24 h treatment period using the Olympus IX73 inverted microscope (Olympus, Tokyo, Japan). The captured images were analyzed using the cellSens Dimensions v.1.8. Software (Olympus, Tokyo, Japan). The highest (1:16) and the lowest (1:1) dilutions were selected for this evaluation. 

### 2.8. Biocompatibility Testing in 3D Reconstructed Human Epidermis

The biocompatibility of ARA was assessed using the EpiDerm™ Skin Irritation Test for Medical Device Extracts fabricated by MatTek Corporation (Ashland, MA, USA). Negative control (NC) for this assay was Dulbecco’s phosphate-buffered saline (DPBS), while positive control (PC) was 1% sodium dodecyl sulfate (SDS). Dulbecco’s phosphate-buffered saline (DPBS), sodium dodecyl sulfate (SDS), and assay medium (EPI-100-NMM) were supplied by the manufacturer (MatTek Corporation, Ashland, MA, USA).

The test was conducted according to the manufacturer’s recommendations [37] as follows: (i) the tissues were removed from the 24-well plate, removing all traces of agarose to facilitate their examination; (ii) the tissues were then placed in 6-well pre-filled plates with 0.9 mL medium and placed in the incubator for 1 h; (iii) after this time, the tissues were moved to pre-filled wells with fresh medium and incubated overnight; (iv) the next day, a volume of 100 μL of the undiluted medical device extracts (artificial saliva) was applied, together with NC and PC, and the tissues were incubated for 18 h; (v) after 18 h, the MTT assay was applied. The tissues were washed and placed in plates of 24 pre-filled gouges with 0.3 mL/well MTT solution and then placed in the incubator for 3 h; (vi) after MTT incubation time, the tissues were transferred to 24-well plates prefilled with 2 mL isopropanol (MTT-100-EXT extractant solution) per plate; (vii) the plate was sealed with parafilm and placed in a plate shaker for 2 h; (viii) after this period, 2 μL × 200 μL sample/well per tissue was transferred to a 96-well plate, and absorbance measurements (at 570 nm) were carried out using Cytation 5 (BioTek Instruments Inc., Winooski, VT, USA). The viability values were calculated using the following Equation (1):

(1) $$\mathrm{Viability}\%=\frac{OD_{sample}}{OD{\;}_{NC}}\times 100$$

% viability—(sample OD)/(NC OD) × 100, where OD—optical density and NC—negative control [38].

## 3. Results

### 3.1. Cytotoxicity Assessment 

**Figure 4:**
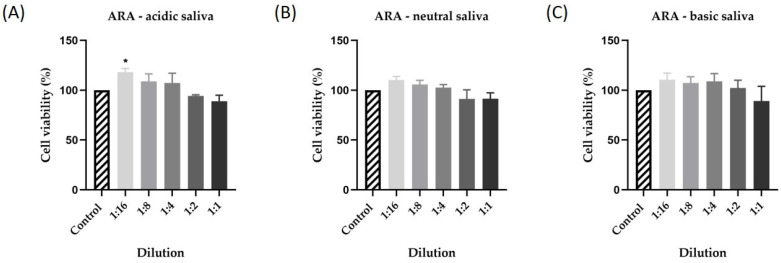
In vitro evaluation of the effect exerted by DM suspended in (**A**) acidic saliva (pH = 3), (**B**) neutral saliva (pH = 7), and (**C**) basic saliva (pH = 10) on the viability of human keratinocytes - HaCaT cells following a 24 h treatment by performing the 3-(4,5-dimethylthiazol-2-yl)-2,5-diphenyltetrazolium bromide (MTT) assay. Five different dilutions were tested (1:16, 1:8, 1:4, 1:2, 1:1). Data are presented as viability percentages (%) normalized to control and expressed as mean values ± SD of three independent experiments performed in triplicate. The statistical differences between the control and the treated group were verified by applying the one-way ANOVA analysis followed by Dunett’s multiple comparisons post-test (* *p* < 0.1).

The first step in verifying the biocompatibility and toxic potential of ARA was to determine the effect of the three types of saliva on human keratinocytes after 24 h of stimulation. The results showed low activity for all three types of saliva tested. Thus, in Figure 3, there is a very small decrease in cell viability depending on the concentration tested and the salivary pH. Consequently, the lowest cell viability was recorded for acid saliva at a 1:1 dilution at 88% (Figure 4A). For the other samples tested, cell viability did not decrease below 91.55% for neutral saliva (Figure 4B) and 89.25% for basic saliva (Figure 4C).

**Figure 5:**
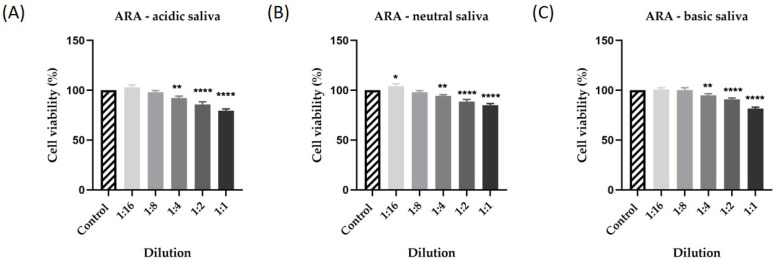
In vitro evaluation of the effect exerted by DM suspended in (**A**) acidic saliva (pH = 3), (**B**) neutral saliva (pH = 7), and (**C**) basic saliva (pH = 10) on the viability of HaCaT cells following a 72 h treatment by performing the MTT assay. Five different dilutions were tested (1:16, 1:8, 1:4, 1:2, 1:1). Data are presented as viability percentages (%) normalized to control and expressed as mean values ± SD of three independent experiments performed in triplicate. The statistical differences between the control and the treated group were verified by applying the one-way ANOVA analysis followed by Dunett’s multiple comparisons post-test (* *p* < 0.1, ** *p* < 0.01, and **** *p* < 0.0001).

Regarding the cell viability recorded after stimulation of human keratinocytes for 72 h, there was a slight decrease in the percentage of viable cells compared to the viability recorded at 24 h. Thus, in Figure 5, there is a decrease in cell viability, dependent on concentration and salivary pH. In the case of the acidic saliva in which ARA was maintained, the lowest cell viability was recorded, approximately 79% (Figure 5A). In the case of neutral and basic saliva, cell viability was slightly influenced at the highest dilutions tested, the percentage of viable cells being about 85% in the case of neutral saliva (Figure 5B) and about 82% in the case of basic saliva (Figure 5C). 

### 3.2. Cell Morphology and Confluence Evaluation

**Figure 6:**
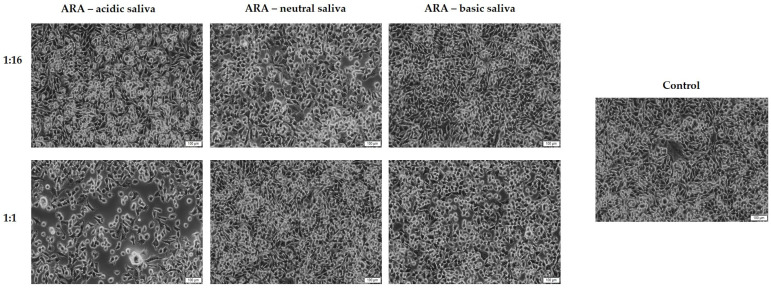
Morphology and confluence of HaCaT cells following the 24 h stimulation with ARA suspended in acidic, neutral, and basic artificial saliva. The highest (1:16) and the lowest (1:1) dilutions were selected for this evaluation. The scale bars represent 100 µm.

As a component of the toxicological profile of ARA, a microscopic evaluation of the HaCaT cells’ morphology and confluence was conducted. According to Figure 6, at the dilution of 1:16, no significant changes in the confluence of HaCaT cells were detected when compared to control cells. However, the cells’ treatment with DM at the dilution of 1:1 led to a reduction in the confluence of the cells. The most prominent effect was noticed following the treatment with ARA suspended in acidic saliva.

### 3.3. Biocompatibility Testing in Reconstructed Human Epidermis

**Figure 7:**
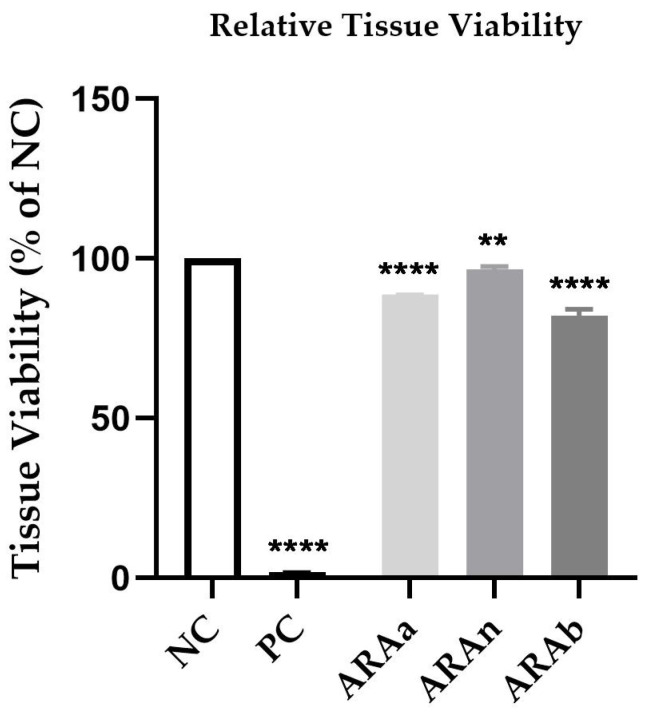
Viability percentage of EpiDerm skin model inserts (EPI-200 SIT) at 18 h post-treatment with the test sample (ARAa, ARAn, and ARAb) at the dilution of 1:1. One-way ANOVA analysis and Dunett’s post-test were performed to determine the statistical differences between sample-treated inserts and negative control-treated inserts (** *p* < 0.01; **** *p* < 0.0001). Positive control (PC) is 1% SDS, while the negative control (NC) is DPBS.

The treatment of EpiDerm insert with ARAa, ARAn, and ARAb 1:1 led to a decrease in viability to 88.77%, 96.68%, and 82.20%, respectively. The treatment with 1% SDS (PC) significantly decreased the tissue viability to 1.95%. According to the manufacturer’s protocol, a sample is considered an irritant if the tissue viability post-treatment is lower than 50% compared to NC. Hence, the tested ARA can be classified as non-irritant, since the viability exceeded 50% regardless of the type of saliva used for its preparation (Figure 7).

## 4. Discussion

There is no formal definition of malocclusion as a disease, but the fact that it is a problem among preschoolers has made it a major health issue. The main features of orthodontic treatment are the low age of patients and a fairly long duration of treatment, which can exceed 2–3 years [39]. Due to the multiple side effects associated with classical orthodontic therapy, such as the loss of hard dental tissues and the appearance of dental sensitivity, recent studies have focused on the development of new orthodontic alternatives for the treatment of malocclusion [40]. The main concern of the orthodontic specialist is patient safety, especially due to the fact that in orthodontic practice most patients are children or adolescents. For this reason, a very important aspect is the biocompatibility of the orthodontic appliance used [41].

Based on these considerations, the purpose of the present study was to evaluate the safety and biocompatibility of an acrylic removable appliance developed by Family Dental Clinic in collaboration with Docs Lab from Timisoara. For this purpose, the medical device was kept in artificial saliva for ten days, and then the saliva was tested in vitro, using both the 2D model—the human keratinocyte cell line (HaCaT)—and a 3D model (3D reconstructed human epidermis).

The removable appliance was made in Docs Lab from Timisoara using materials such as polymethylmethacrylate (PMMA, Orthocryl^®^) for the base plate, and the metal clamps were made of hard wire with round spring, LEOWIRE C0400-07 and C0400-08. Classification according to chemical structure of the materials used to make removable orthodontic appliances includes two important groups: (i) methacrylate acrylic resins; and (ii) Bis-acryl and Bis-GMA composite resins. Of these types of materials, the most widely used is currently polymethylmethacrylate (PMMA) acrylic resins [42]. Polymers have gained the attention of orthodontists due to their mechanical and esthetic properties. Nonetheless, in recent years a warning has been given regarding the release of potentially toxic substances from synthetic polymers. One such substance is bisphenol A, with implications for multiple pathologies, including the development of ovarian cancer [18]. Orthocryl is cold-cured acrylic produced by Dentaurum; it comprises a polymer with large particles that prevent dripping of the acrylic and monomer. It has a longer working time and shorter curing cycle below 40 °C [43]. Due to its ability to prevent the formation of monomers, Orthocryl^®^ is considered a safer material, having no irritant and allergenic potential [44]. In addition, LEOWIRE round spring hard wires C0400-07 and C0400-08 were used to make the metal clamps. Thus, the use of stainless steel alloy prevents allergic side effects [45].

The first step of this study was to prepare the artificial saliva and keep the orthodontic appliance in it for ten days. In this way, it was determined whether potentially toxic substances could be released into the saliva. In addition, to mimic the biological environment as closely as possible, three different pHs of saliva were chosen, one acidic, one neutral, and one basic. After this time, the three types of saliva were applied to the human keratinocytes, and the 3D reconstructed human epidermis. In the oral cavity, under the influence of salivary pH, orthodontic appliances can undergo the process of corrosion and the release of toxic substances and ions. Three pH values have been used in this study, acid, neutral, and basic, because salivary pH can fluctuate greatly in the oral cavity as a result of diet, which then impacts the biocompatibility of the dental appliance [46]. The ten days for maintaining the medical device in saliva were chosen due to previous studies that revealed that the main metal ions, Co, Cr, and Ni, are released from the orthodontic appliances in the first days of treatment [47]. As part of our previous study to verify the biocompatibility of two types of orthodontic devices, artificial saliva was used successfully, allowing us to conduct a preliminary study with medical devices for ten days [35]. A similar artificial saliva environment has been used in other orthodontic studies. Thus, Schiff et al. conducted a comparative study on three orthodontic appliances made of stainless steel, cobalt–chromium, and titanium. Researchers have analyzed the effect of mouthwash on appliance corrosion, noting major effects on stainless steel and titanium brackets [48].

Further, the experimental study consisted of verifying the toxicological profile and biocompatibility of the medical device by testing the three types of saliva in which the device was kept for ten days. Saliva was tested in five different dilutions (1:16, 1:8, 1:4, 1:2, and 1:1) in human keratinocytes that were monitored for cell viability for a period of 24 and 72 h. The results showed that none of the saliva types exerted a pronounced cytotoxic effect at 24 and 72 h. However, the lowest cell viability was recorded for saliva with acidic pH used in a 1:1 dilution, the viability value being approximately 89% at 24 h and approximately 79% at 72 h. Additionally, in this case there was a change in confluence and a decrease in the number of cells observed after 24 h. A similar study was previously conducted to determine the biocompatibility of two types of orthodontic appliances. The obtained results indicated that the use of this type of artificial saliva and the human keratinocyte cell line are suitable for the preliminary evaluation of the toxicological profile of orthodontic devices [35]. An important aspect in performing cytotoxicity studies is choosing the type of cell line. Thus, according to a recent study on the types of cell lines suitable for evaluating the biocompatibility of orthodontic appliances, human keratinocytes (HaCaT) are among the most suitable in vitro models for this type of assessment [49]. HaCaT has an increased proliferation rate but nevertheless retains the characteristics of the original tissue, making it one of the most widely used cell lines in scientific research [50]. In a similar study of human keratinocytes, several types of acrylic resin used in orthodontics were evaluated. The results indicated that none of the 28 types of material caused a marked decrease in the viability of human keratinocytes [51]. Tanis et al. [52] conducted a study on the cytotoxicity of a polymerized acrylic resin under different heat cycles, two hard acrylic-based coating materials and a long-term soft material. The results obtained revealed that the materials obtained under conditions similar to those tested in the present study (by polymerization under pressure at a temperature of 40–45 °C) have a good biocompatibility suitable for clinical use. The increased biocompatibility of these types of materials can be explained by the pressure polymerization process that eliminates the formation of porosity. If the material has porosity, the release of toxic substances is easier to achieve [53]. Therefore, in the present study, the lack of porosity of the material may have been a contributing factor to its lack of toxicity. In addition to the flat base, which was made of polymethylmethacrylate, ARA is also composed of metal clamps made of LEOWIRE round spring hard wire C0400-07 and C0400-08 (stainless steel alloy). Previous studies have studied the effect of pH on the release of metal ions from orthodontic alloys [54]. Thus, Kao et al. [55] have shown that stainless steel has a cytotoxic effect when maintained in saliva with acidic pH. Galeotti et al. [56] also evaluated the effect of stainless steel on human keratinocytes after maintaining it in acidic pH saliva and obtained similar results regarding the cytotoxicity of the material. As outlined, these studies support the results obtained in the present study and explain the slight decrease in cell viability observed in artificial acid saliva. 

Finally, to provide a better picture of the toxicological profile of ARA, an in vitro 3D model represented by the 3D reconstructed human epidermis was used on which the EpiDerm ™ Skin Irritation Test for Medical Device Extracts method was applied. The highest dilutions previously tested in human keratinocytes were selected for this assay. The results showed that none of the three samples showed an irritating effect, the lowest viability being approximately 82% in the case of artificial saliva with basic pH, and the best biocompatibility was observed in the case of saliva with neutral pH, where cell viability was very close to that of negative control (approximately 97%). The use of 3D models in vitro allows the evaluation of toxicity and irritant effect, providing results with increased in vivo reproducibility [57]. Previously, in a study by Vannet et al. [58], the toxicity of several types of orthodontic archwires was assessed using the in vitro 3D model. The results indicated that of the three types of orthodontic wires, stainless steel had the lowest toxicity, being considered biocompatible. To the best of our knowledge, the cytotoxic effect of polymerized acrylic resin on 3D reconstructed human epidermis has not been tested to date. 

A major goal of fundamental in vitro research is to obtain as complex information as possible about the behavior of a medical substance or device in vitro so that it can later be correlated with in vivo effects [59]. Hence, it is imperative to include as many parameters as possible in in vitro studies that may impact the substance’s behavior in vivo. Thus, in the present study, the parameter considered was salivary pH. The pH of the oral cavity can vary depending on the food ingested, various pathologies, such as gastrointestinal reflux, or the presence of microorganisms [60]. Thus, by varying the pH, orthodontic devices can yield different substances with desired effects at the systemic level [61]. For this reason, it was decided to use three types of saliva with different pHs so that the results could be correlated with the possible behavior that the device could have in vivo. 3D reconstructed human epidermis is also an alternative to the in vivo Draize rabbit eye and skin irritation tests [62]. This method has been standardized to evaluate the toxicity of extracts of medical devices [63]. With these factors in mind, the present study sought to create the most accurate in vivo conditions so that the results could be applied to future studies.

The main limitation of the present study is the small sample size. In future studies, it is necessary to evaluate the biocompatibility of ARA in several cell lines. It is also necessary to prolong the time of immersion of ARA in artificial saliva, as well as to prolong the time of exposure of cells to artificial saliva. Further studies are needed to evaluate the release of potentially toxic ions and substances from ARA as well as their mechanism of action at the cell line. Another limitation of the study is that in vitro studies do not take into account certain in vivo factors, such as diet or microflora of patients.

## 5. Conclusions

The acrylic removable appliances (ARA) has a good stability in the biological environment, due to the materials from which it is made. As proof of the stability of the ARA, the results of in vitro tests indicate that it is suitable for clinical use. Following in vitro evaluation of the human keratinocyte cell line, the artificial saliva in which ARA was maintained did not cause a significant decrease in cell viability and did not produce significant morphological changes. However, a slight decrease in cell viability was observed in the case of artificial saliva with acidic pH. In addition, in the in vitro 3D model, 3D reconstructed human epidermis, at the largest dilutions of artificial saliva, did not produce toxic or irritating effects. In conclusion, the results of the study support the hypothesis that ARA has a good stability in the biological environment without the release of toxic substances.

## Data Availability

All of the data are available within the manuscript.
